# Relationship between premature loss of primary teeth with oral hygiene, consumption of soft drinks, dental care, and previous caries experience

**DOI:** 10.1038/srep21147

**Published:** 2016-02-26

**Authors:** Sandra Aremy López-Gómez, Juan José Villalobos-Rodelo, Leticia Ávila-Burgos, Juan Fernando Casanova-Rosado, Ana Alicia Vallejos-Sánchez, Salvador Eduardo Lucas-Rincón, Nuria Patiño-Marín, Carlo Eduardo Medina-Solís

**Affiliations:** 1 Academic Area of Dentistry of Health Sciences Institute at Autonomous University of Hidalgo State, Pachuca, Mexico; 2 Epidemiology Department, ISSSTE, Culiacan, Mexico; 3 School of Dentistry of Autonomous University of Sinaloa, Culiacan, México; 4 Health Systems Research Centre at National Institute of Public Health, Cuernavaca, Mexico; 5 School of Dentistry of Autonomous University of Campeche, Campeche, México; 6 Advanced Studies and Research Center in Dentistry “Dr. Keisaburo Miyata” of School of Dentistry at Autonomous University State of Mexico, Toluca, México; 7 Clinical Research Laboratory of Dental Sciences Doctorate Program at Autonomous University of San Luis Potosí, San Luis Potosí, Mexico

## Abstract

We determine the relationship between premature loss of primary teeth and oral hygiene, consumption of soft drinks, dental care and previous caries experience. This study focused on 833 Mexican schoolchildren aged 6–7. We performed an oral examination to determine caries experience and the simplified oral hygiene index. The dependent variable was the prevalence of at least one missing tooth (or indicated for extraction) of the primary dentition; this variable was coded as 0 = no loss of teeth and 1 = at least one lost primary tooth. The prevalence of at least one missing tooth was 24.7% (n = 206) (95% CI = 21.8–27.7). The variables that were associated with the prevalence of tooth loss (p < 0.05) included: the largest number of decayed teeth (OR = 1.11), the largest number of filled teeth (OR = 1.23), the worst oral hygiene (OR = 3.24), a lower frequency of brushing (OR = 1.60), an increased consumption of soda (OR = 1.89) and use of dental care (curative: OR = 2.83, preventive: OR = 1.93). This study suggests that the premature loss of teeth in the primary dentition is associated with oral hygiene, consumption of soft drinks, dental care and previous caries experience in Mexican schoolchildren. These data provide relevant information for the design of preventive dentistry programs.

The primary dentition, besides serving an obvious chewing function, acts as a guide for the eruption of permanent teeth. The primary dentition also stimulates the growth of the jaw and aids in digestion and phonation. Primary dental arches form the basis for the proper development of permanent dental arches^1^. Several conditions that occur during the development of the primary dentition and the transition to the permanent dentition that are considered quite normal and predictable. However, premature loss of primary teeth can result in some negative consequences in both dentitions and cause an imbalance in the normal development of the stomatognathic system. Dental caries has been identified as one of the leading causes of tooth loss in children around the world^2^,^3^. In this sense, dental extraction is the most common form of dental treatment in developing countries despite the significant progress made in recent years in dentistry^4^. The results of studies realized in various countries have reported prevalence of premature loss of primary teeth ranging from 8.5% in Venezuela^3^ to 16.5% in India^5^ to 51% in Saudi Arabia^6^.

In Mexico, as in the international field, dental caries is a significant public health problem among preschoolers and schoolchildren^7^,^8^; they represent the leading cause of tooth loss in children^1^. As of now, few studies have focused on premature loss of primary teeth and the factors responsible for this prevalence^3^,^5^,^9^. Here, we accordingly focus on determining the relationship between premature loss of primary teeth with oral hygiene, consumption of soft drinks, dental care and previous caries experience in Mexican schoolchildren.

## Materials and Methods

### Study design and sample selection

We employed a secondary analysis of a cross-sectional study to evaluate a variety of conditions of oral health among schoolchildren attending any one of the 18 elementary schools in the city of Navolato, Sinaloa, Mexico. Oral health was evaluated in terms of dental caries, premature tooth loss, oral hygiene, use of dental health services, and dental pain, among other aspects^10^,^11^.

During the first phase of the study, we talked with health and education-sector representatives of the city. Next, mothers/guardians of the children were invited to participate in the study. Individuals who accepted the invitation were asked to sign a letter of informed consent. The inclusion criteria included: a) enrollment in a primary school, b) an age of 6 or 7 years, and c) a parent or guardian agreeing to participate in the study. The exclusion criteria included: a) a condition that would preclude oral inspection and b) refusal to receive a clinical examination. The final sample consisted of 833 schoolchildren.

### Variables included in the study and data collection

To collect sociodemographic, socioeconomic and behavioral information, the mothers/guardians of the children were asked to fill out a questionnaire.

All of the subjects included in the study were examined clinically by one of three examiners. Prior to collecting the data, we carried out a standardization exercise on the criteria used in the study via a pilot study. The dependent variable in the study was the loss of at least one tooth of the primary dentition and coded as: 0 = if there was no tooth loss and 1 = if there was at least one primary tooth lost. As recommended by the World Health Organization (WHO), missing primary teeth should be considered only if the subject is at an age when normal exfoliation would not be a sufficient explanation for the absence of teeth^12^. Early loss was classified by subtracting 12 months from the chronological table of eruption of permanent teeth proposed by Hurme, as has been suggested by a variety of authors^13^. For the detection of dental caries, the WHO criteria^12^ and Pitts d1 lesions^14^ was used. The criterion “plaque” from the Simplified Oral Hygiene Index (S-OHI) was used to determine the level of oral hygiene in the schoolchildren^15^.

The independent variables of the children included in the study were: age, sex, age at commencement of tooth brushing, frequency of tooth brushing, age at which bottle feeding ceased, consumption of sugar from sweets, frequency of soft drink consumption, and type of dental care in the past year. Furthermore, information on oral hygiene and the number of decayed and filled teeth was collected. The independent variables of the parental figures included in the study were: age, occupation and education. We also collected socioeconomic variables such as family size, status of health insurance, school type and car ownership in household. Caries experience was evaluated using the decay, missing and filled teeth index (dmft index)^12^.

To build the variable “sugar consumption,” we used six variables that explored the weekly frequency of consumption of sugars in solution, semi-solid and solid substances. Next, using principal components analysis with polychoric correlation matrices^16^, we used the first component, which explained 52.0% of the variability in the schoolchildren’s consumption of sugars. The new variable generated was categorized into tertiles – the first tertile represented the group with the lowest consumption of sugars, and the third tertile represented the group with the highest consumption of sugars. The weekly consumption of soft drinks was considered to be another independent variable and categorized as 0 = ≤1 soft drink consumed per day and 1 = >1 soft drink consumed per day. Moreover, socioeconomic status (SES) was determined using the schooling and occupation of both parents; the first component generated explained 57.5% of the variability in SES. The variable was categorized into tertiles for analysis; the first tertile refers to the lowest socioeconomic level, and the third tertile refers to the highest socioeconomic level.

### Statistical analysis

We obtained the frequencies and percentages of each category of qualitative variables. We calculated measures of central tendency and dispersion for the continuous variables. For the bivariate analysis, we used the χ^2^, Mann-Whitney, Kruskal-Wallis and Spearman correlation tests according to the measuring scale of the variables. Finally, we fit a multivariate binary logistic regression model to estimate the strength of association between primary tooth loss and the independent variables, which is expressed as odds ratios with 95% confidence intervals (95% CI). We also report the p values that were considered statistically significant (p value < 0.05). For the construction of the final model, we considered the variables that were in the bivariate analysis with a statistical significance of p < 0.25. We performed a variance inflation factor analysis test to detect and avoid multicollinearity between the independent variables. We used the specification error test to verify the assumption that the *logit* of the response variable is a linear combination of the independent variables. After fixing the main effects, we tested the interactions, but none proved to be significant at p < 0.15. Finally, to assess the overall fit of the model, we used the goodness of fit test^17^. The confidence intervals of both the bivariate and multivariate analysis were calculated with robust standard Huber-White errors, which can yield valid estimates even in cases of groups being correlated. Since the data were from children attending elementary schools that shared common characteristics (i.e., the data were clustered), we assumed that observations within these clusters would be correlated and that observations between clusters would not be correlated^18^. We performed the analysis using Stata 11.0 statistical software.

We applied “estimation by regression” to the missing data^19^; the variables on which imputations were made included the age of fathers (n = 32) and mothers (n = 3), SES (n = 45) and soda consumption (n = 22).

### Ethical aspects

This study was carried out according to the general health law in research and the scientific principles of the Declaration of Helsinki. Written consent was obtained from the parents/guardians of the participating children. The protocol was approved by the ethics committee review board of the National Institute of Public Health where one of the authors earned his Master’s degree in Public Health.

## Results

We included a total of 833 schoolchildren, of which 52.1% were female and 54.9% were 7 years old. The descriptive data of the subjects included in the study are shown in Table 1. The prevalence of at least one missing tooth (or indicated for extraction) was 24.7% (95% CI = 21.8–27.7), and the average number of missing teeth was 0.47 ± 1.08. Among the 206 schoolchildren with missing teeth, we observed a total of 381 missing teeth; 101 and 280 had been extracted and indicated for extraction, respectively.

Table 2 presents the bivariate results of the logistic regression; the variables that were significantly associated included the mother’s age, the number of decayed and filled teeth, the presence of plaque, frequency of brushing, consumption of sugars and soft drinks and type of dental care in the past year. We note that an older the mother’s age act as a protective factor for the loss of primary teeth; on the contrary, the highest number of decayed and filled teeth and the greatest amount of plaque were risk indicators for tooth loss. Similarly, a lower frequency of tooth brushing, an increased consumption of sugars from sweets, soda consumption and having used dental services in the last year were positively associated with tooth loss.

Based on our multivariate logistic regression model, after adjusting for age and sex (Table 3), the prevalence of primary teeth loss was positively associated (p < 0.05) with the largest number of decayed teeth (OR = 1.11, 95% CI = 1.03–1.20), the largest number of filled teeth (OR = 1.23, 95% CI = 1.13–1.34), a lower frequency of tooth brushing (OR = 1.60, 95% CI = 1.18–2.15), and an increased consumption of soft drinks (OR = 1.89, 95% CI = 1.13–3.16). The worst oral hygiene condition was one of the factors that we found to most significantly predict tooth loss (OR = 3.24, 95% CI = 1.65–6.39); curative dental care services were also associated with tooth loss (OR = 2.83, 95% CI = 2.30–3.49). We additionally found, to a lesser degree, that the use of preventive dental services was associated with tooth loss (OR = 1.93, 95% CI = 1.24–3.00).

## Discussion

Our results reveal that prevalence of tooth loss in the primary dentition was high (24.7%); this loss is associated with clinical and behavioral variables. We also showed that tooth loss is a serious public health problem in this population.

The prevalence of loss of primary teeth has been studied only rarely. However, studies carried out of Mexico reveal prevalence ranging from 8.5–51.0%^3^,^5^,^6^; the prevalence in this study was 24.7%. Preserving primary teeth until normal exfoliation is one of the most important factors in preventive and interceptive dentistry. Although in recent decades there has been a decrease in the frequency of oral diseases, it is likely that dental care or treatment of a teething child is considered to be low priority for parents and guardians because of the “temporary” nature of these teeth. However, the consequences of tooth loss include pain and suffering, and these consequences are also associated with high costs in terms of both health systems and the family’s economy^20^,^21^. Therefore, it is critical to design preventive dentistry programs that are cost effective and widespread that include a healing component aimed at preventing premature tooth loss in children.

Oral hygiene can be measured via two indicators: the presence of plaque (objectively) and self-reported tooth brushing (subjectively). The presence of dentobacterial plaque, which is composed of bacteria and toxins and is the main etiological factor of tooth decay, is the major cause why children’s teeth are removed^22^. Systematic tooth brushing is believed to mechanically remove dentobacterial plaque and suppress its activity and stop the development of the initial injury. Tooth brushing is accordingly considered to be one of more cost-effective measures to remove dentobacterial plaque and prevent the development of dental caries and subsequent tooth loss^9^,^23^,^24^. This study includes both indicators, and we found that both the simplified oral hygiene index and the frequency of tooth brushing were predictors of loss of primary teeth.

This study reveals, similar to in the findings of studies of dental caries^25^, the existence of an association between tooth loss and soft drink consumption by children. The high content of sugar in sweetened drinks has been associated with the presence of dental caries. Decay is caused by acids produced mainly from the interaction of specific bacteria with these sugars. Although bacteria produce acids that are considered to be a cause of caries, the bacteria themselves do not produce sufficient acid to demineralize tooth enamel. Therefore, it is the presence of sugars or cooked foods rich in starch that increase the production of acids^26^. For this reason, it is necessary that the parental figures modify their children’s unhealthy behaviors at an early age. Furthermore, the implementation of public policies to reduce the consumption of sweetened beverages, such as increasing taxes on these products, may help to be prevent tooth decay and tooth loss^27^.

Studies have revealed that past experience of dental caries is associated with the subsequent appearance of new lesions^25^,^28^. In a study conducted in Turkey^9^, it was demonstrated that the presence of untreated caries (the “decayed” component of the dmft index) and the number of teeth treated due to caries (the “filled” component of the dmft index) are associated with premature loss of primary molars. These findings are consistent with the results of this study; we found that both components of the dmft index are risk indicators for premature loss of primary teeth. This same relationship was recovered by Virtanen *et al.*^29^, although in permanent dentition. These authors found that subjects who had earlier restorations had a higher risk of their future teeth being restored due to dental caries. It has also been documented that, over time, these restored teeth are extracted the most often^30^.

We found an increase in the likelihood of loss of primary teeth among children who used oral health services (more so with curative services but also with preventive services). The use of oral health services having a negative effect on oral health is consistently observed in Mexico; people only seek out services when their oral health is already compromised^31^; this fact is particularly true in the case of curative services. Although Sheiham^32^ noted that oral health services have had little impact on reducing dental caries; in Mexico, public health institutions are limited in these services (fillings, extractions and preventive care). This situation does not include specialty care. Furthermore, Mexico is characterized by heterogeneity in terms of the quality and delivery of health care. Budget constraints within public institutions render specialized treatments (such as pediatric dentistry, endodontics, etc.) unavailable in public coverage, forcing patients to directly pay for such care in private dental services (i.e.,, which constitutes a barrier to access to this type of service^33^). Therefore, maintaining primary teeth is complicated.

The results of this study suggest that the premature loss of teeth in the primary dentition is associated with oral hygiene, consumption of soft drinks, and previous experience of dental caries in Mexican schoolchildren. These data provide relevant information for the design of programs of preventive dentistry. It is important to take action to reduce the premature loss of primary teeth, and we must disseminate information to parents and guardians about the importance of preserving the primary dentition to eliminate the myth that primary teeth are only temporary.

## Additional Information

**How to cite this article**: López-Gómez, S. A. *et al.* Relationship between premature loss of primary teeth with oral hygiene, consumption of soft drinks, dental care, and previous caries experience. *Sci. Rep.* **6**, 21147; doi: 10.1038/srep21147 (2016).

## Figures and Tables

**Table 1:** Distribution of the children’s characteristics included in the study.

Variable	Mean ± sd	Limits
Father’s age (years)	36.09 ± 6.30	22–65
Mother’s age (years)	33.13 ± 5.44	20–54
Number of decayed teeth	4.94 ± 3.00	0–16
Number of filled teeth	0.61 ± 1.67	0–16
Oral Hygiene (S-OHI)	1.10 ± 0.32	0–2.5
	**n**	**%**
Sex		
Boys	399	47.9
Girls	434	52.1
Children’s age		
6 years	376	45.1
7 years	457	54.9
Family size (number of children)		
0–1	389	46.7
2–3	397	47.7
>4	47	5.6
Started toothbrushing		
≤2 years of age	207	24.8
>2 years of age	626	75.2
Toothbrushing frequency		
At least 1/day	414	49.7
Less than 1/day	419	50.3
Age at which bottle feeding ceased		
Not use or up to two years	681	81.7
After two years	152	18.3
Consumption of sugar from sweets		
Low	370	44.4
Regular	193	23.2
High	270	32.4
Sugared soft drink consumption		
≤1 times/day	751	90.2
>1 times/day	82	9.8
Type of dental care in the past year		
Without dental care	326	39.1
Curative care	348	41.8
Preventive care	159	19.1
Type of insurance		
Public insurance	562	67.5
Not insured	134	16.1
Private insurance	137	16.4
Socioeconomic status		
First tertil (lowest)	277	33.2
Second tertil	278	33.4
Third tertil (highest)	278	33.4
School type		
Private	157	18.8
Public	676	81.2
Car ownership		
No	383	46.0
yes	450	54.0

**Table 2:** Bivariate analysis between the loss of primary teeth and the variables included in the study.

Variable	OR 95% CI	P value
Father’s age (years)	0. 98 (0.96–1.00)	0.055
Mother’s age (years)	0.97 (0.94–1.00)	0.044
Number of decayed teeth	1.10 (1.03–1.19)	0.006
Number of filled teeth	1.21 (1.12–1.30)	< 0.001
Oral Hygiene (S-OHI)	3.78 (2.03–7.06)	< 0.001
Sex		
Boys	1*	
Girls	1.19 (0.91–1.55)	0.202
Children’s age		
6 years	1*	
7 years	1.20 (0.89–1.62)	0.231
Family size (number of children)		
0–1	1*	
2–3	0.92 (0.67–1.26)	0.611
>4	0.58 (0.25–1.34)	0.207
Started toothbrushing		
≤2 years of age	1*	
>2 years of age	1.34 (0.94–1.92)	0.105
Toothbrushing frequency		
At least 1/day	1*	
Less than 1/day	1.49 (1.16–1.91)	0.002
Age at which bottle feeding ceased		
Not use or up to two years	1*	
After two years	1.47 (0.99–2.19)	0.058
Consumption of sugar from sweets		
Low	1*	
Regular	1.10 (0.78–1.56)	0.594
High	1.43 (1.04–1.96)	0.03
Sugared soft drink consumption		
≤1 times/day	1*	
>1 times/day	2.00 (1.25–3.20)	0.004
Type of dental care in the past year		
Without dental care	1*	
Curative care	3.20 (2.61–3.93)	< 0.001
Preventive care	2.05 (1.34–3.12)	0.001
Type of insurance		
Public insurance	0.84 (0.49–1.42)	
Not insured	1*	0.512
Private insurance	0.83 (0.58–1.18)	0.305
Socioeconomic status		
First tertil (lowest)	1*	
Second tertil	1.07 (0.64–1.79)	0.783
Third tertil (highest)	1.01 (0.65–1.57)	0.947
School type		
Private	1*	
Public	0.91 (0.65–1.28)	0.6
Car ownership		
No	1*	
yes	0.91 (0.63–1.34)	0.658

*Reference category.

**Table 3:** Multivariate logistic regression analysis for loss of primary teeth.

Variable	OR 95% CI	P value
Number of decayed teeth	1.11 (1.03–1.20)	0.006
Number of filled teeth	1.23 (1.13–1.34)	< 0.001
Oral Hygiene (S-OHI)	3.24 (1.65–6.39)	0.001
Toothbrushing frequency		
At least 1/day	1*	
Less than 1/day	1.60 (1.18–2.15)	0.002
Sugared soft drink consumption		
≤1 times/day	1*	
>1 times/day	1.89 (1.13–3.16)	0.015
Type of dental care in the past year		
Without dental care	1*	
Curative care	2.83 (2.30–3.49)	< 0.001
Preventive care	1.93 (1.24–3.00)	0.004

^*^Reference category.

Note: Model adjusted for the variables in the table as well as by age and sex.

95% CI estimated with robust standard errors (*cluster* school).

Goodness-of-fit test: Pearson x^2^ (688) = 687.31, p=0.5002.

Link test (specification error): predictor=0.002; predictor^2^ = 0.117.
